# Enabling In Vivo Optical Imaging of an Osmium Photosensitizer by Micellar Formulation

**DOI:** 10.3390/pharmaceutics14112426

**Published:** 2022-11-10

**Authors:** Drashti Shah, Menitte Eroy, John Fakhry, Azophi Moffat, Kevin Fritz, Houston D. Cole, Colin G. Cameron, Sherri A. McFarland, Girgis Obaid

**Affiliations:** 1 Department of Bioengineering, University of Texas at Dallas, Richardson, TX 75080, USA; 2 Department of Chemistry and Biochemistry, University of Texas at Arlington, Arlington, TX 76019, USA

**Keywords:** photodynamic therapy, osmium photosensitizers, hypoxia, micellar formulation, luminescence imaging, phosphorescence imaging

## Abstract

Osmium (Os)-based photosensitizers (PSs) exhibit unique broad, red-shifted absorption, favoring PDT activity at greater tissue depths. We recently reported on a potent Os(II) PS, *rac-*[Os(phen)_2_(IP-4T)](Cl)_2_ (ML18J03) with submicromolar hypoxia activity. ML18J03 exhibits a low luminescence quantum yield of 9.8 × 10^−5^ in PBS, which limits its capacity for in vivo luminescence imaging. We recently showed that formulating ML18J03 into 10.2 nm DSPE-mPEG_2000_ micelles (Mic-ML18J03) increases its luminescence quantum yield by two orders of magnitude. Here, we demonstrate that Mic-ML18J03 exhibits 47-fold improved accumulative luminescence signals in orthotopic AT-84 head and neck tumors. We show, for the first time, that micellar formulation provides up to 11.7-fold tumor selectivity for ML18J03. Furthermore, Mic-ML18J03 does not experience the concentration-dependent quenching observed with unformulated ML18J03 in PBS, and formulation reduces spectral shifting of the emission maxima during PDT (variance = 6.5 and 27.3, respectively). The Mic-ML18J03 formulation also increases the production of reactive molecular species 2–3-fold. These findings demonstrate that micellar formulation is a versatile and effective approach to enable in vivo luminescence imaging options for an otherwise quenched, yet promising, PS.

## 1. Introduction

Photodynamic therapy (PDT) is a minimally invasive modality used in a variety of cancer and non-cancer indications. Its therapeutic action relies on the selective accumulation of a photosensitizer (PS) molecule in diseased tissue followed by the light-activated production of cytotoxic and biomodulatory reactive molecular species (RMS). Such RMS molecules include, but are not limited to, singlet oxygen, hydroxyl radicals, hydrogen peroxide, superoxide anions and peroxynitrite anions [1]. A distinguishing feature of PDT is the ability to simultaneously perform multiparametric optical imaging to personalize and guide treatment [2]. This is made possible by the intrinsic fluorescence or phosphorescence properties of many PSs in pre-clinical and clinical development. In vivo luminescence imaging is often used to estimate PS accumulation in tissue to inform the optimal injected dose and ideal timing for photoactivation to maximize tissue selectivity. In addition, photobleaching of the luminescence signal has been used pre-clinically and clinically to guide PDT dosimetry implicitly [2,3,4,5]. Here, implicit dosimetry refers to the measurement of the indirect consequences of PDT that imply that an effective PDT dose has been administered, whereas explicit dosimetry refers to more direct measurements of the applied PDT dose [5]. Explicit PDT dose metrics include the light fluence and fluence rate, PS tissue concentration, oxygen concentration, and singlet oxygen concentration to model the applied PDT dose [6]. Regardless, luminescence imaging of the PS itself plays a central role in PDT dosimetry and personalization.

Metal-based PSs are emerging as highly versatile, potent, and promising PDT agents [7]. Of note, our ruthenium-based photosensitizer (TLD1433) has successfully completed phase I in clinical trials for non-muscle invasive bladder cancer (NMIBC) [7] and is currently in a Phase 2 study. We also recently reported a hypoxia-active Os(II)-based PS, (*rac-*[Os(phen)_2_(IP-4T)](Cl)_2_, referred to as ML18J03) with potent submicromolar phototherapeutic efficacy under hypoxic conditions [8]. Certain Os(II) complexes such as ML18J03 and its close relatives have the potential for eliciting tumor tissue phototoxicity at greater depths owing to their longer wavelength absorption windows. In addition, these Os(II) PSs are designed specifically to exhibit prolonged excited state lifetimes for increased RMS generation, with both visible and NIR wavelengths of light [8,9]. As a result, ML18J03 has a very high singlet oxygen quantum yield (95% from the lowest lying ^3^ILCT state). We have shown that ML18J03 exhibits excellent in vivo tolerability with maximum tolerated doses (MTD) exceeding 200 mg/kg [9]. Despite these attractive features, ML18J03 is not suitable for in vivo optical imaging because its luminescence is almost completely suppressed in an aqueous solution due to aggregation-induced quenching [10]. Novel approaches are therefore required to harness the luminescence imaging capabilities of ML18J03 as well as other promising Os(II) complexes.

**Figure 1:**
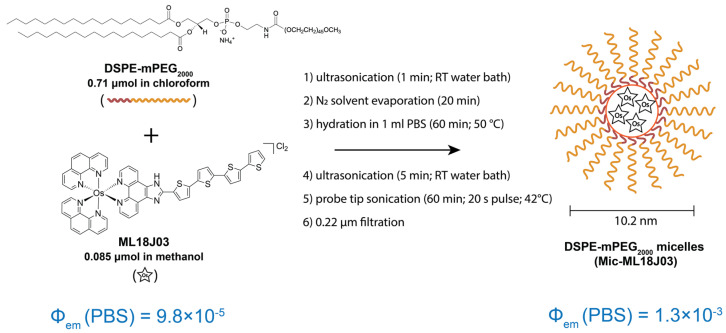
Diagrammatic representation of the formulation of ML18J03 into DSPE-mPEG_2000_ micelles in order to increase their luminescence quantum yield in aqueous environments and enable in vivo optical imaging.

A variety of delivery systems exist that have been demonstrated to effectively carry hydrophobic, aggregation-prone PS molecules [1,11]. Among these, micelles show promise due to their relatively low toxicity, high biocompatibility, small diameters that favor tumor penetration, and prolonged circulation times [12,13,14]. Tseng et al. reported fluorinated Ce6-loaded PFFA polymeric micelles that show promising therapeutic results in vitro [15]. P3H2 polymeric micelles, with a peptide targeting HER-2 receptor in breast cancer cells, were also found to be a promising drug delivery vehicle in 3D cell models [16]. Along with this, pH/glutathione (GSH) responsive nano-prodrug micelles demonstrate in vivo efficacy for MRI-guided tumor PDT [17]. We previously showed that DSPE-mPEG_2000_ micelles are an efficient and robust platform that stably encapsulate a hydrophobic conjugate of the PS benzoporphyrin derivative and cholesterol, even when liposomes fail to do so [18]. Our recent study has shown that ML18J03, when formulated in 10.2 nm DSPE-mPEG_2000_ micelles, exhibits low dark toxicity, submicromolar EC_50_ values in hypoxia, retains its photoactivity in normoxia, and provides a significant reduction in inter-assay variability of ML18J03 [10]. Of particular significance to this study, the formulation of ML18J03 into DSPE-mPEG_2000_ micelles increases its luminescence quantum yield in PBS from 9.8 × 10^−5^ to 1.3 × 10^−3^, Figure 1 [10]. As such, in this study, we explore the capability of micellar formulation to enable luminescence imaging of ML18J03 in vivo in orthotopic AT-84 head and neck tumors and assess their tumor selectivity following intravenous administration.

## 2. Materials and Methods

### 2.1. Preparation of Free ML18J03 and Mic-ML18J03

ML18J03 was synthesized as previously described [8]. Aqueous Mic-ML18J03 preparations were obtained by formulating the PS in DSPE-mPEG_2000_ micelles according to our previously published protocol [10]. Briefly, 0.085 μmol of ML18J03 (1 mg/mL stock in methanol) was mixed with 0.71 μmol DSPE-mPEG_2000_ (25 mg/mL stock in chloroform; NOF America Coorporation, White Plains, NY, USA) to provide 11 mol% PS with respect to total lipid content. The mixture was sonicated for 1 min in an ultrasonic water bath at room temperature and the solvent was evaporated using nitrogen gas flow. The dry residue was then hydrated in 1 mL Dulbecco’s phosphate buffered saline (PBS, pH 7.4; Corning, Corning, NY, USA) at 50 °C for 1 h. The hydrated mixture was then sonicated for 5 min in an ultrasonic water bath at room temperature. Using a probe tip sonicator (Fisher Scientific (Hampton, NH, USA) 20 kHz Model 120 Sonic Dismembrator), the hydrated mixture was then sonicated for a total of 1 h at 42 °C using 20 s on–40 s off cycles to form micelles. After cooling to room temperature, the micelles were filtered using 0.22 μm polyethersulfone filters (MilliporeSigma, Burlington, MA, USA) and the ML18J03 concentration was measured using UV-visible spectrophotometry (ε_436 nm_ = 6.2 × 10^4^/M/cm in acetonitrile). Micelles were also characterized using a Zetasizer Pro (Malvern Panalytical, Northampton, MA, USA) Dynamic Light Scattering system and stored at 4 °C in the dark.

Aqueous solutions of unformulated ‘free ML18J03’ were prepared by first dissolving ML18J03 in dimethylsulfoxide (DMSO) (Fisher Scientific (Hampton, NH, USA) as a 10 mM stock. Free ML18J03 was then diluted to 100 μM in PBS to a final DMSO content of 1% and used without storage.

### 2.2. Spectroscopic Measurements of ML18J03 Preparations

Absorption spectra of the free ML18J03 (1% DMSO in PBS) and Mic-ML18J03 (in PBS) were measured using a Thermo Evolution 350 Spectrophotometer between 200 nm and 800 nm. Luminescence emission spectra of the free ML18J03 (1% DMSO in PBS) and Mic-ML18J03 (in PBS) were measured using a Horiba (Kyoto, Japan) Fluorlog Fluorometer from 600 nm to 800 nm using excitation at 448 nm.

### 2.3. Quantification of Photogenerated Reactive Molecular Species (RMS)

Singlet oxygen production was measured using the fluormetric probe Singlet Oxygen Sensor Green (SOSG; Fisher Scientific (Hampton, NH, USA) and the colorimetric probe Anthracene-9,10-dipropionic acid (ADPA; Fisher Scientific (Hampton, NH, USA). Production of hydroxyl radicals and peroxynitrite was measured using the fluorometric probe Hydroxyphenyl Fluorescein (HPF; Fisher Scientific (Hampton, NH, USA).

For the SOSG singlet oxygen measurement assay, free ML18J03 and Mic-ML18J03 samples were prepared at 5 μM concentrations either in 1% DMSO in PBS or pure PBS, respectively. Samples were placed in a 96-well plate in 100 μL aliquots and a 10 μL SOSG probe (50 μM stock in PBS) was added to each well. Fluorescence emission was measured using the Tecan (Männedorf, Switzerland) Infinite M Plex Plate Reader (λ_Exc_ = 460 nm; λ_Emi_ = 530 nm) before and after photoirradiation with 420 nm LED light (Biolambda (São Paulo, Brazil) 420 nm LED box; irradiance of 46.08 mW/cm^2^) at 0.2 J/cm^2^ increments until a maximum fluence of 1.0 J/cm^2^.

Anthracene-9,10-dipropionic acid was also used to measure singlet oxygen generation. The free ML18J03 and Mic-ML18J03 samples were prepared at 5 μM concentrations as described above and placed in a 96-well plate in 100 μL aliquots. ADPA (5 μL; 6 mM stock in methanol) was added to each sample. Absorbance was measured at 378 nm using the Tecan Infinite M Plex Plate Reader. Samples were then irradiated with 420 nm LED light (Biolambda 420 nm LED box; irradiance of 46.08 mW/cm^2^) at 0.5 J/cm^2^ increments until a maximum fluence of 3.0 J/cm^2^.

Hydroxyl radicals and peroxynitrite anions were measured using the HPF probe. The samples were prepared at 5 μM concentrations and placed in a 96-well plate in 100 μL aliquots as described above. HPF (20 μL, 200 μM stock in PBS) was added to each sample. Fluorescence emission was measured using the Tecan Infinite M Plex Plate Reader (λ_Exc_ = 460 nm; λ_Emi_ = 530 nm) before and after irradiation. Samples were irradiated with 420 nm LED light (Biolambda 420 nm LED box; irradiance of 46.08 mW/cm^2^) at 0.5 J/cm^2^ increments until a maximum fluence of 5.0 J/cm^2^.

### 2.4. Photobleaching of Free ML18J03 vs. Mic-ML18J03

Free ML18J03 and Mic-ML18J03 were prepared in 1% DMSO in PBS and pure PBS, respectively, at 5 μM PS equivalent as described above. Luminescence spectra of the preparations were measured using a Horiba (Kyoto, Japan) Fluorlog Fluorometer ((λ_Exc_ = 448 nm; λ_Emi_ = 600–800 nm) before and after 15 J/cm^2^ increments of 420 nm LED light until a maximum fluence of 90 J/cm^2^. Photobleaching was measured as a decrease in luminescence emission intensities following photoactivation.

### 2.5. In Vivo Tumor Imaging

Luminescence imaging was performed in vivo in an orthotopic murine model of head and neck cancer. AT-84 cells were a gift from Dr. Michael Story (University of Texas Southwestern Medical Center). AT-84 cells (2 × 10^6^ cells in 50 µL of sterile PBS) were orthotopically implanted in male C3H/HeJ mice (Jackson Laboratories, Bar Harbor, ME) in the head and neck region according to an adaptation of a previously described procedure [19]. At 22 d after implantation, the tumors reached an approximate size of 5–8 mm. Mice were then intravenously injected with 30 mg/kg of free ML18J03 (1% DMSO in PBS) or Mic-ML18J03 (in PBS) through the tail vein. The tumor region was then longitudinally imaged using the IVIS Lumina Series III in vivo imaging system (λ_Exc_ = 440 nm; λ_Emi_ = 710 nm; PerkinElmer, Waltham, MA, USA) at 1, 3, 6, 19, 24, and 48 h after administration. Autofluorescence signals for each animal prior to administration of free ML18J03 or Mic-ML18J03 were subtracted, and the corrected in vivo luminescence signals have been used throughout the study.

### 2.6. Confocal Imaging of Tumor Cyrosections

Following the 48 h imaging, the mice were humanely euthanized and the tumors were harvested. The tumors were then bisected with a razor blade and cryopreserved in OCT (Tissue-Plus™ O.C.T. Compound; Fisher Scientific) at −80 °C. The frozen tumors were then cryosectioned at 20 µm slices using the Cryostat-Leica CM1860. The tumor cryosections were then defrosted to room temperature and imaged using the Olympus FV3000RS Confocal Laser Scanning Microscope (UPLSAPO 10× objective; Excitation laser = 405 nm; Detector Gain = 2.25; Detector Offset = −2.0; Detector Voltage = 490.0; Olympus, Center Valley, PA, USA). For image analysis, five randomized regions of interest (ROIs) were taken of the slices across the entire tumor cross-section and, using FIJI image analysis software, the mean luminescence value was measured. Background signals of the glass slide without tissue were used to correct the mean intensities of ML18J03 in the tissue.

## 3. Results and Discussion

We have previously reported that the unformulated, free ML18J03 used in this study has EC_50_ values ranging from 7 × 10^−4^ μM to 0.0434 μM in SK-MEL-28 melanoma cells, following broadband visible light excitation, with considerable inter-assay variability due to its propensity to aggregate [10]. The liposomal and micellar formulations of ML18J03 from our previous study have EC_50_ values of 98.1 × 10^−4^ μM and 49.1 × 10^−4^ μM, respectively, in SK-MEL-28 cells following broadband visible light excitation. Using related cyclometalated Ru(II) complexes, we previously reported EC_50_ values ranging from 0.142 μM to 0.258 μM in SK-MEL-28 and HL60 cells following broadband visible light excitation [20]. Using [Os(phen)2(IP-nT)]Cl_2_ complexes related to ML18J03, with varying thiophene chain lengths (nT), we also reported EC_50_ values following broadband visible light excitation ranging from 1 μM to 3 μM for Os-nT = 0 to 2, and 0.153 μM to 18 × 10^−6^ μM for Os-3T and Os-4T, respectively [8]. However, the low water solubility and extensive aggregation can be significant limitations for these compounds. Our ruthenium-based photosensitizer (TLD1433) currently being evaluated in clinical trials exhibits an EC_50_ value of 1.9 × 10^−4^ μM in SK-MEL-28 [7]. Other clinically approved PSs, such as Verteporfin (Visudyne; Bausch + Lomb, Laval, Canada) and Porfimer Sodium (Photofrin; Pinnacle Biologics, Bannockburn, IL) exhibit EC_50_ values of 0.61–1.21 µM and 4.5 µM, respectively [21,22]. To put the PDT efficacy of Mic-ML18J03 into perspective, a comparison with other reported micelles previously used for PDT can be made. C225-conjugated chlorin e6-loaded polymeric micelles have exhibited an EC_50_ value of 0.173 μM in A431 cells [23]. Furthermore, Protoporphyrin IX-lipid micelles exhibited an EC_50_ value of 35.6 μM in HeLa cells [24]. A folate-mediated and pH-responsive chidamide-bound micelle system encapsulating the PS pyropheophorbide-*a* also exhibited an EC_50_ value of 0.062 μM in A2780 cells [25]. While direct comparisons of EC_50_ do not take into consideration the wavelength, fluence and irradiance of photoexcitation, it is still evident that ML18J03 is a highly potent PS that is active in hypoxia and in normoxia. Micellar formulation of ML18J03 also improves water solubility and offers a more robust PDT response with significantly lower inter-assay variability. As such, this study proceeds to explore the ability of micellar formulation to significantly enhance the in vivo luminescence imaging options for ML18J03.

### 3.1. Luminscence Spectroscopy

We previously reported that ML18J03 exhibits a markedly low luminescence quantum yield in PBS of 9.8 × 10^−^^5^ [10]. By formulating ML18J03 into PEG-modified DPPC liposomes and DSPE-mPEG_2000_ micelles, the luminescence quantum yield in PBS was increased to 1.1 × 10^−^^3^ and 1.3 × 10^−^^3^, respectively. Compared to liposomal ML18J03, the micellar ML18J03 formulation (Mic-ML18J03) exhibited a slightly larger phototherapeutic margin in cellular assays [10], a higher luminescence quantum yield, and a 10-fold smaller diameter favoring greater tumor tissue penetration. As such, we investigated whether the enhanced the luminescence quantum yield for Mic-ML18J03 would translate to improved in vivo luminescence imaging of ML18J03 in an orthotopic AT-84 murine head and neck tumor model. In this study, all aqueous samples of free ML18J03 were prepared by first dissolving the agent in DMSO as a co-solvent and diluting the sample in PBS at a final DMSO concentration of 1%. Free ML18J03 samples were used immediately after preparation to minimize aggregation over time.

**Figure 2:**
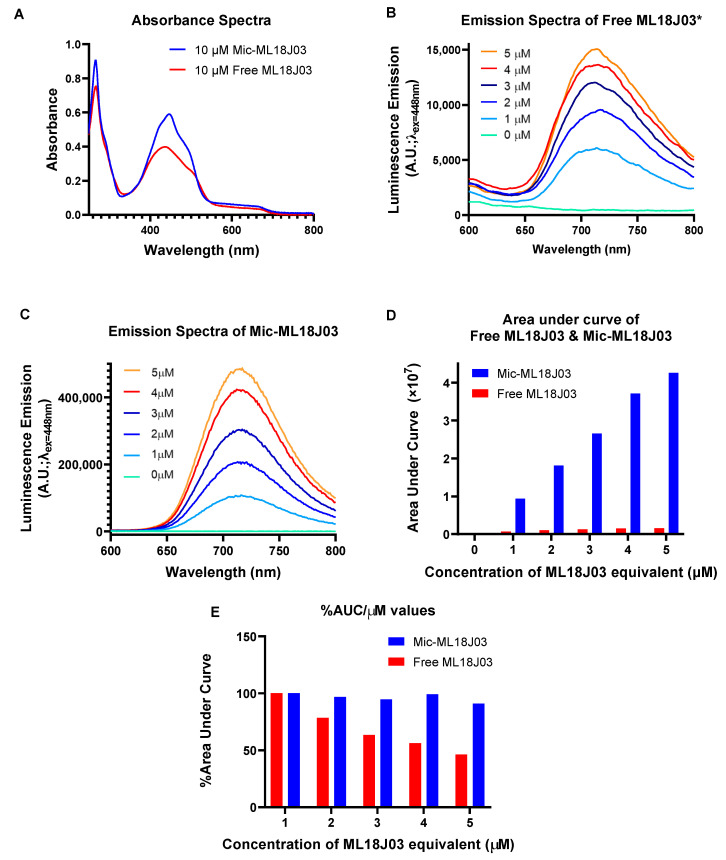
(**A**) Absorbance spectra of free ML18J03 (1% DMSO in PBS) and Mic-ML18J03 (in PBS) at 10 μM PS equivalent. Raw luminescence emission spectra (λ_Exc_ = 448 nm, λ_Emi_ = 600–800 nm) of 1–5 μM of (**B**) free ML18J03 in 1% DMSO in PBS* and (**C**) Mic-ML18J03 in PBS. (**D**) Area under the curve analyses for the luminescence emission spectra of 1–5 μM of free ML18J03 and Mic-ML18J03. (**E**) Percent-area under the curve analyses of 1–5 μM of free ML18J03 and Mic-ML18J03 with respect to the lowest concentration of 1 μM ML18J03 equivalent, demonstrating the concentration-dependent static quenching only observed with free ML18J03. (* Smoothing curve analysis applied with a factor of 10 for clarity in (**B**). Only raw spectral data were used for further analysis).

Free ML18J03 and Mic-ML18J03 were prepared as described and their absorbance and luminescence spectra were recorded (Figure 2A–C). Free ML18J03 (1% DSMO in PBS) exhibited 10 nm blue-shifted absorbance maxima at 437 nm, with respect to Mic-ML18J03 in PBS (Figure 2A). The absorbance maximum of free ML18J03 was also dampened by 32%, as compared to Mic-ML18J03, which is indicative of sample aggregation. These observations support our previously published dynamic light scattering data that shows that free ML18J03 is present as aggregates with a mean diameter of 1.6 µm when suspended in 1% DMSO in PBS, whereas Mic-ML18J03 is present as stable micelles with a mean diameter of 10.2 nm when dispersed in PBS [10].

At all concentrations tested, Mic-ML18J03 exhibited higher emission intensities as demonstrated by Area Under the Curve (AUC) analyses of the luminescence spectra (Figure 2D). The greatest difference was observed at 5 μM ML18J03 equivalent, where Mic-ML18J03 exhibits a 27-fold increased luminescence, as compared to the free ML18J03. Figure 2E shows the relative values for the luminescence emission of both samples that have been normalized to the lowest concentration tested (1 μM ML18J03 equivalent) to depict any concentration dependence of the emission. Above 1 µM the emission of free ML18J03 is diminished with increasing concentration. This suggests that free ML18J03 experiences concentration-dependent aggregation and static quenching above 1 μM ML18J03 equivalent. In contrast, the Mic-ML18J03 does not experience concentration-dependent static quenching at any of the concentrations tested, suggesting that ML18J03 does not aggregate when entrapped in the micelles. The significance of these results is that Mic-ML18J03 appears to be a more reliable preparation when using luminescence measurements for semi-quantitative analyses of ML18J03 biodistribution and tissue micro-distribution.

### 3.2. Photogenerated Reactive Molecular Species (RMS)

**Figure 3:**
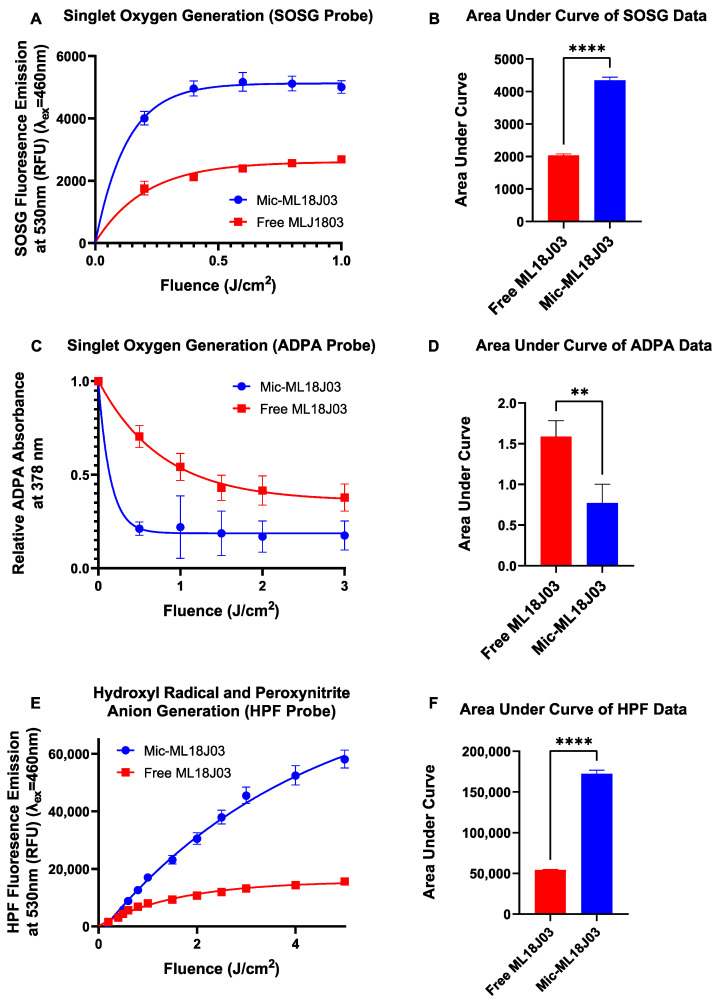
(**A**,**B**) Production of singlet oxygen by free ML18J03 and Mic-ML18J03 measured using the fluorescent probe Singlet Oxygen Sensor Green (SOSG; λ_Exc_ = 460 nm, λ_Emi_ = 530 nm). (**C**,**D**) Singlet oxygen generation by free ML18J03 and Mic-ML18J03 measured using the colorimetric probe anthracene dipropionic acid (ADPA) at 378 nm absorbance. (**E**,**F**) Hydroxyl radical and peroxynitrite generation by free ML18J03 and Mic-ML18J03 measured using the fluorescent probe hydroxyphenyl fluorescein (HPF). (All of the data is presented as mean ± standard error; statistical significance was calculated using a two-tailed *t*-test, **: *p* ≤ 0.01, ****: *p* ≤ 0.0001).

Although the objective of micellar formulation of ML18J03 is to enable in vivo luminescence imaging, it is imperative to ensure that ML18J03 retains its ability to generate RMS when formulated for efficient PDT. We have recently shown that Mic-ML18J03 retains its therapeutic PDT efficacy in vitro under both normoxic and hypoxic conditions, and in fact, produces a more robust response with lower variability between biological replicates [10]. We have also shown that Mic-ML18J03 exhibits a singlet oxygen quantum yield of 2.5 × 10^−^^3^ in PBS, whereas the singlet oxygen quantum yield of free ML18J03 was undetectable in an aqueous solution. Here, we probe the efficiency of photogeneration (420 nm excitation) of various RMS species after micellar formulation, namely singlet oxygen (using SOSG and ADPA probes), hydroxyl radicals (using HPF probe) and peroxynitrite anions (using HPF probe). We have previously used these probes to quantify the photogeneration of RMS from various lipid nanoformulations of PS molecules [26]. Mic-ML18J03 exhibited a 2-fold increase in singlet oxygen generation with respect to free ML18J03, as determined by both the SOSG and ADPA probes (Figure 3A–D). Furthermore, Mic-ML18J03 also demonstrated a 3.2-fold increase in hydroxyl radical and peroxynitrite anion generation with respect to free ML18J03, as measured by the HPF probe (Figure 3E,F). As such, Mic-ML18J03 demonstrates favorable photophysical properties, with respect to free ML18J03, in addition to its enhanced luminescence, thereby providing further motivation for its use in future in vivo image-guided PDT studies. Such future studies will also include investigations into the impact of micellar formulation on in vivo generation of RMS in tumor tissue and healthy tissue. These can include in situ singlet oxygen phosphorescence imaging or the in vivo use of biocompatible fluorescent RMS probes.

### 3.3. Assessment of Photobleaching

**Figure 4:**
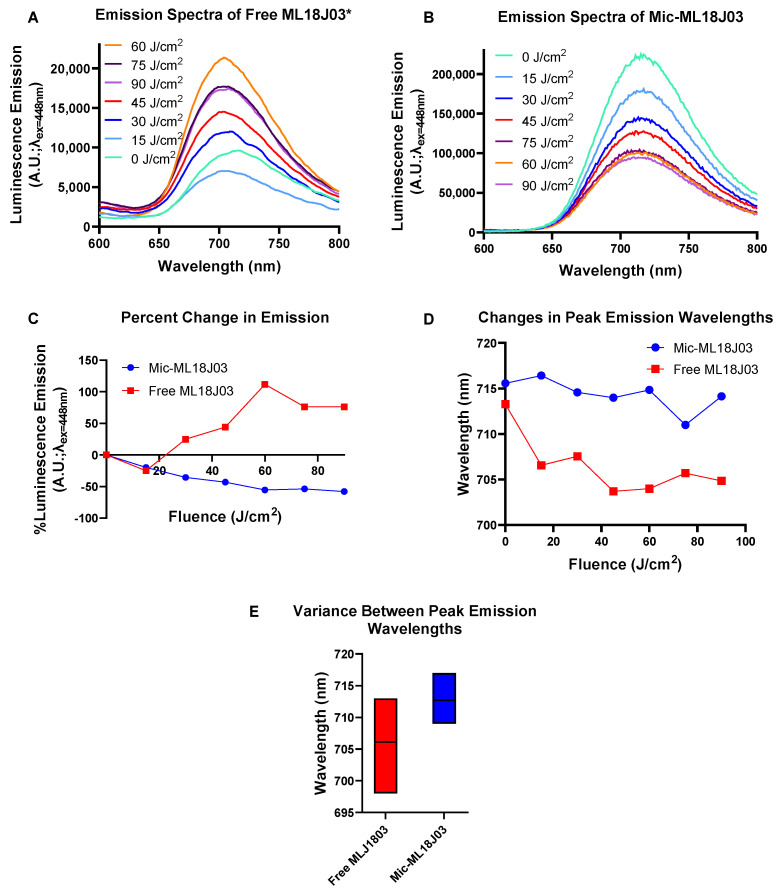
Raw luminescence emission spectra of free ML18J03 in 1% DMSO and PBS (**A**) and Mic-ML18J03 in PBS (**B**; λ_Exc_ = 448 nm, λ_Emi_ = 600–800 nm) upon photoirradiation using 420 nm light at an irradiance of 46.08 mW/cm^2^. (**C**) Relative changes in the peak luminescence emission values upon photoirradiation. (**D**) Shifts in the wavelengths of emission maxima of free ML18J03 and Mic-ML18J03 upon photoirradiation. (**E**) Min-max plot depicting the variations in peak luminescence emission wavelength for both free ML18J03 (variance = 27.26) and Mic-ML18J03 (variance = 6.48) during photoirradiation from 0–90 J/cm^2^. (Smoothing curve analysis applied with a factor of 10 to (**A**)).

Photobleaching of PSs following photoactivation diminishes their luminescence emission, which is a result of oxidative self-degradation while diseased tissue is also sensitized [18]. The efficiency of photobleaching is typically associated with a PS’s ability to sensitize tumor tissue. Photobleaching has been widely used as an implicit guide to estimate and customize PDT dosimetry [6]. As such, photobleaching of free ML18J03 and Mic-ML18J03 was measured following excitation with 420 nm light, where ML18J03 exhibits the highest molar absorptivity, and photobleaching would thus be expected to be the strongest. A clear inverse relationship between the light fluence and luminescence emission of Mic-ML18J03 was observed. (Figure 4A,B), which resulted in a ~50% reduction in luminescence emission after 90 J/cm^2^ of 420 nm light (Figure 4C). However, it appears that the rate of photobleaching is reduced as the fluence is increased. This is especially pronounced between 75 J/cm^2^ to 90 J/cm^2^. The rate of photobleaching is dependent on a number of factors including oxygenation. It is likely that the ML18J03 molecules closest to the surface of the micelles are more rapidly bleached than those at the core of the micelles, as a result of immediate oxygen availability. However, this hypothesis must be systematically studied in future mechanistic studies exploring the role of oxygenation of photobleaching of Mic-ML18J03.

The same fluence-dependent effect on luminescence intensities did not hold for free ML18J03, where emission initially decreased by ~25% and then increased by up to 100% after 60 J/cm^2^ of 420 nm light (Figure 4C). The inconsistency observed following the excitation of free ML18J03 could be attributed to dynamic aggregation and de-aggregation in response to photoirradiation, which was further supported by the spectral shifting of the emission band. By contrast, Mic-ML18J03 exhibited a much lower variance in spectral shifting (variance = 6.48) with respect to free ML18J03 (variance = 27.26; Figure 4D,E). For accurate in vivo monitoring of the PS during the course of treatment, it is extremely important to reduce spectral shifting in the emission maxima of PSs during the PDT procedure. Taken together, these results demonstrate how the micellar formulation of ML18J03 provides more reliable measurements of photobleaching to monitor implicit PDT dosimetry.

### 3.4. In Vivo Imaging

**Figure 5:**
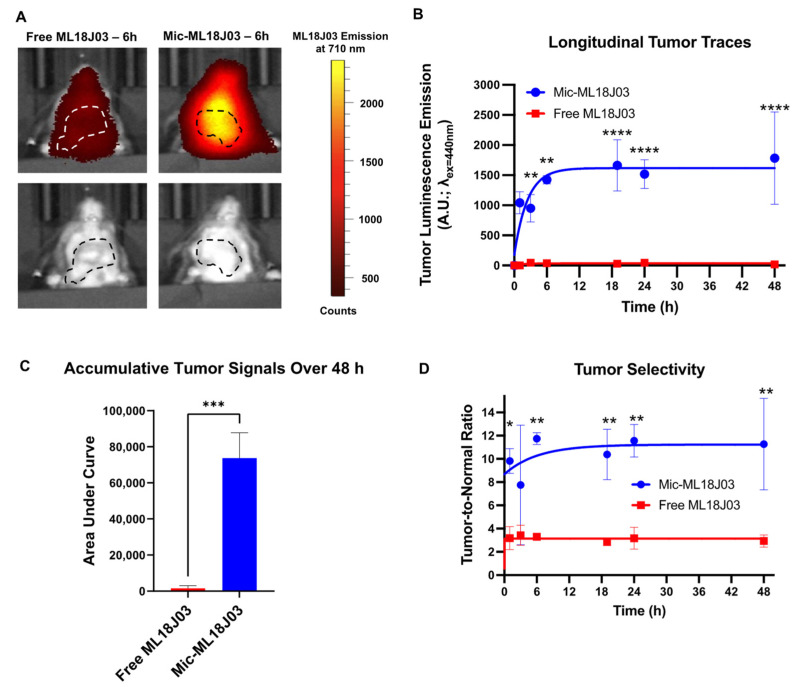
(**A**) Representative images of orthotopic in vivo AT-84 tumors in C3H/HeJ mice 6 h following intravenous administration of free ML18J03 or Mic-ML18J03 (30 mg/kg); (λ_Exc_ = 440 nm, λ_Emi_ = 710 nm). Quantification of the longitudinal (**B**) and accumulative (**C**) in vivo luminescence emission signals of free ML18J03 or Mic-ML18J03 up to 48 h following administration. (**D**) Tumor selectivity of free ML18J03 or Mic-ML18J03 with respect to nearby healthy tissue. (All luminescence emission signals have been corrected for tissue autofluorescence for each respective animal. All of the data is mean ± standard deviation; statistical significance was calculated using a One-Way ANOVA test (**A**,**D**) and a two-tailed *t*-test (**D**). *: *p* ≤ 0.05, **: *p* ≤ 0.001, ***: *p* ≤ 0.001, ****: *p* ≤ 0.0001).

The primary objective of this study was to assess if the micellar formulation of ML18J03 could enable in vivo luminescence imaging of the PS, which was otherwise not possible. As such, we performed longitudinal in vivo luminescence imaging of orthotopic AT-84 tumors following intravenous administration of 30 mg/kg ML18J03 equivalent of Mic-ML18J03 or free ML18J03. Autofluorescence signals for each animal were subtracted from the respective luminescence signals. Over the 48 h duration of in vivo imaging, tumors in mice administered with Mic-ML18J03 showed a 47-fold increase in accumulative luminescence emission, as compared to mice administered with free ML18J03 which remained almost undetectable at all time points (Figure 5A–C). This difference is assumed to be due to differential tumor tissue uptake between the free ML18J03 and Mic-ML18J03. Considering that micellar formulation improves the water solubility of ML18J03 and simultaneously provides a stealth PEG-coated nanoscale vehicle, it will likely improve its pharmacokinetic profile by prolonging its plasma half-life, reducing its renal clearance and increasing the efficiency of passive tumor accumulation. Future studies are aimed at using elemental analysis of Os in the tumor tissue, blood and organs to study the impact of micellar formulation on pharmacokinetics in comparison with free ML18J03 which cannot be imaged accurately using luminescence.

Importantly, tumor selectivity was found to range from ~8–12-fold with respect to nearby healthy tissue when using Mic-ML18J03, with the highest selectivity at 6 h after administration (Figure 5D). Conversely, tumor selectivity of free ML18J03 was only ~3-fold with respect to nearby healthy tissue at all timepoints. This improved tissue selectivity is an added advantage of using DSPE-mPEG micellar formulations to carry ML18J03 and motivate future studies that explore its phototherapeutic efficacy in the clinic. Although tumor selectivity here is achieved by passive extravasation and accumulation in tumor tissue, future studies will also explore the impact of molecular targeting of Mic-ML18J03 on tumor selectivity, pharmacokinetics and phototherapeutic efficacy.

**Figure 6:**
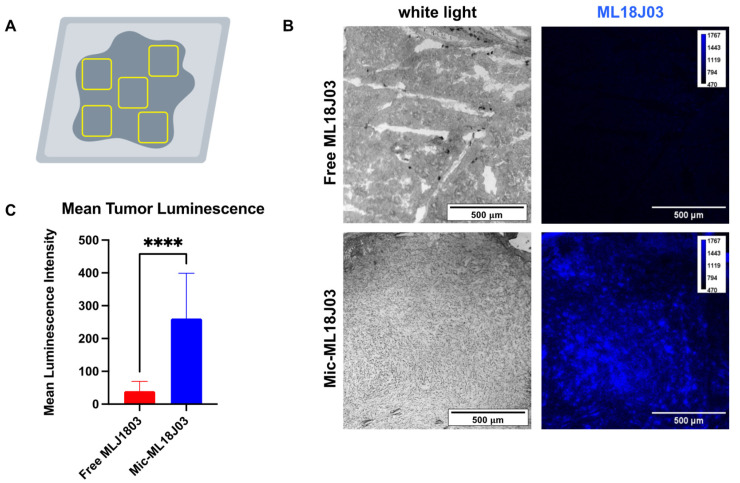
(**A**) Diagrammatic representation of the randomly sampled regions of interest (ROIs) across the entire tumor cross-section for mice administered with either Mic-ML18J03 or Free ML18J03. Representative white light and luminescence images (**B**; false color blue) and quantitative analysis (**C**) of tumor sections obtained from bisected AT-84 tumors 48 h following intravenous administration of free ML18J03 or Mic-ML18J03. (Images were processed using ImageJ; scale bar and calibration bar are represented) (All of the data is mean ± standard deviation; *n* = 3 (5 ROIs per tumor); statistical significance was calculated using a non-parametric two-tailed *t*-test, ****: *p* ≤ 0.0001).

At 48 h following administration, mice were humanely euthanized, tumors were harvested and bisected, and cryosectioned to image ML18J03 in the tumor cores. Five random Regions of Interest (ROIs) were imaged across the sections from each tumor in mice administered with both Free-ML18J03 and Mic-ML18J03 (Figure 6A). Tumor sections from mice administered with Mic-ML18J03 exhibited a 6.5-fold increase in luminescence emission, as compared to the tumor sections from mice administered with free ML18J03 (Figure 6B,C; blue false color assignment). The significance of this observation lies in the fact that Mic-ML18J03 is capable of penetrating tumor tissue. Future studies will quantify the time dependence of extravasation into various solid tumors of different sizes.

## 4. Conclusions

Optical imaging is an integral component of PDT. Highly potent emerging PSs, such as ML18J03, which are also strongly hydrophobic, are incapable of fully benefiting from the luminescence imaging capabilities that typically support pre-clinical and clinical PDT. This is due to their extremely low luminescence emission quantum yields in aqueous environments. In this study, we show that the micellar formulation of ML18J03 (Mic-ML18J03) is a robust and facile, yet highly efficient method for harnessing the in vivo luminescence imaging capabilities of ML18J03. While increasing the luminescence of sensitizers such as ML18J03 can result in a decrease in singlet oxygen quantum yield, we show here that micellar formulation in fact increases the efficiency of RMS production. It is apparent that micellar formulation prevents radiationless relaxation in ML18J03 while enhancing both the luminescence quantum yield and the singlet oxygen quantum yield in aqueous environments. Furthermore, ML18J03, only when formulated in micelles, becomes traceable for up to 48 h in orthotopic AT84 head and neck tumors with significantly higher tumor selectivity than the free PS. By presenting these results, we provide a unique route for providing optical imaging capabilities for otherwise dark PSs, thereby allowing them to benefit from luminescence image-guided PDT approaches. Considering the simplicity of the approach and the translatability of lipid micellar systems, the strategy we present here is likely to enable in vivo luminescence imaging of a wide range of highly quenched metal and non-metal-based PS molecules. The promising findings of this study warrant further exploration of the micellar formulation of ML18J03 for image-guided PDT of various solid and disseminated tumors in vivo.

## References

[1] Bhandari C., Guirguis M., Savan N.A., Shrivastava N., Oliveira S., Hasan T., Obaid G. (2021). What NIR Photodynamic Activation Offers Molecular Targeted Nanomedicines: Perspectives into the Conundrum of Tumor Specificity and Selectivity. Nano Today.

[2] Celli J.P., Spring B.Q., Rizvi I., Evans C.L., Samkoe K.S., Verma S., Pogue B.W., Hasan T. (2010). Imaging and Photodynamic Therapy: Mechanisms, Monitoring, and Optimization. Chem. Rev..

[3] Anbil S., Rizvi I., Celli J.P., Alagic N., Hasan T. (2013). A Photobleaching-Based PDT Dose Metric Predicts PDT Efficacy over Certain BPD Concentration Ranges in a Three-Dimensional Model of Ovarian Cancer. Opt. Methods Tumor Treat. Detect. Mech. Tech. Photodyn. Ther. XXII.

[4] James N.S., Cheruku R.R., Missert J.R., Sunar U., Pandey R.K. (2018). Measurement of Cyanine Dye Photobleaching in Photosensitizer Cyanine Dye Conjugates Could Help in Optimizing Light Dosimetry for Improved Photodynamic Therapy of Cancer. Mol. A J. Synth. Chem. Nat. Prod. Chem..

[5] Pogue B.W., Elliott J.T., Kanick S.C., Davis S.C., Samkoe K.S., Maytin E.V., Pereira S.P., Hasan T. (2016). Revisiting Photodynamic Therapy Dosimetry: Reductionist & Surrogate Approaches to Facilitate Clinical Success. Phys. Med. Biol..

[6] Jarvi M.T., Patterson M.S., Wilson B.C. (2012). Insights into Photodynamic Therapy Dosimetry: Simultaneous Singlet Oxygen Luminescence and Photosensitizer Photobleaching Measurements. Biophys. J..

[7] Monro S., Colón K.L., Yin H., Roque J., Konda P., Gujar S., Thummel R.P., Lilge L., Cameron C.G., McFarland S.A. (2019). Transition Metal Complexes and Photodynamic Therapy from a Tumor-Centered Approach: Challenges, Opportunities, and Highlights from the Development of TLD1433. Chem. Rev..

[8] Roque J.A., Barrett P.C., Cole H.D., Lifshits L.M., Shi G., Monro S., von Dohlen D., Kim S., Russo N., Deep G. (2020). Breaking the Barrier: An Osmium Photosensitizer with Unprecedented Hypoxic Phototoxicity for Real World Photodynamic Therapy. Chem. Sci..

[9] Roque J.A., Barrett P.C., Cole H.D., Lifshits L.M., Bradner E., Shi G., von Dohlen D., Kim S., Russo N., Deep G. (2020). Os (II) Oligothienyl Complexes as a Hypoxia-Active Photosensitizer Class for Photodynamic Therapy. Inorg. Chem..

[10] Cole H.D., Eroy M., Roque J.A., Shi G., Guirguis M., Fakhry J., Cameron C.G., Obaid G., McFarland S.A. Establishing a Robust and Reliable Response from a Potent Osmium Based Photosensitizer via Lipid Nanoformulation. Photochem. Photobiol..

[11] Obaid G., Broekgaarden M., Bulin A.-L., Huang H.-C., Kuriakose J., Liu J., Hasan T. (2016). Photonanomedicine: A Convergence of Photodynamic Therapy and Nanotechnology. Nanoscale.

[12] Rubtsova N.I., Hart M.C., Arroyo A.D., Osharovich S.A., Liebov B.K., Miller J., Yuan M., Cochran J.M., Chong S., Yodh A.G. (2021). NIR Fluorescent Imaging and Photodynamic Therapy with a Novel Theranostic Phospholipid Probe for Triple-Negative Breast Cancer Cells. Bioconjugate Chem..

[13] de Morais F.A.P., Goncalves R.S., Campanholi K.S., de Franca B.M., Capeloto O.A., Lazarin-Bidoia D., Balbinot R.B., Nakamura C.V., Malacarne L.C., Caetano W. (2021). Photophysical Characterization of Hypericin-Loaded in Micellar, Liposomal and Copolymer-Lipid Nanostructures Based F127 and DPPC Liposomes. Spectrochim. Acta A Mol. Biomol. Spectrosc..

[14] Gong H., Dong Z., Liu Y., Yin S., Cheng L., Xi W., Xiang J., Liu K., Li Y., Liu Z. (2014). Engineering of Multifunctional Nano-Micelles for Combined Photothermal and Photodynamic Therapy Under the Guidance of Multimodal Imaging. Adv. Funct. Mater..

[15] Tseng T.H., Chen C.Y., Wu W.C., Chen C.Y. (2021). Targeted and Oxygen-Enriched Polymeric Micelles for Enhancing Photodynamic Therapy. Nanotechnology.

[16] Kim Y.J., Ha J.H., Kim Y.J. (2021). Self-Assembled Polymeric Micelles for Targeted Photodynamic Therapy of Human Epidermal Growth Factor Receptor 2 Overexpressing Breast Cancer. Nanotechnology.

[17] Guo H., Liu F., Liu E., Wei S., Sun W., Liu B., Sun G., Lu L. (2022). Dual-Responsive Nano-Prodrug Micelles for MRI-Guided Tumor PDT and Immune Synergistic Therapy. J. Mater. Chem. B.

[18] Obaid G., Jin W., Bano S., Kessel D., Hasan T. (2019). Nanolipid Formulations of Benzoporphyrin Derivative: Exploring the Dependence of Nanoconstruct Photophysics and Photochemistry on Their Therapeutic Index in Ovarian Cancer Cells. Photochem. Photobiol..

[19] Bhandari C., Fakhry J., Eroy M., Song J.J., Samkoe K., Hasan T., Hoyt K., Obaid G. (2022). Towards Photodynamic Image-Guided Surgery of Head and Neck Tumors: Photodynamic Priming Improves Delivery and Diagnostic Accuracy of Cetuximab-IRDye800CW. Front. Oncol..

[20] Sainuddin T., Mccain J., Pinto M., Yin H., Gibson J., Hetu M., McFarland S.A. (2016). Organometallic Ru(II) Photosensitizers Derived from π-Expansive Cyclometalating Ligands: Surprising Theranostic PDT Effects. Inorg. Chem..

[21] Mae Y., Kanda T., Sugihara T., Takata T., Kinoshita H., Sakaguchi T., Hasegawa T., Tarumoto R., Edano M., Kurumi H. (2020). Verteporfin-Photodynamic Therapy Is Effective on Gastric Cancer Cells. Mol. Clin. Oncol..

[22] Roschger C., Verwanger T., Krammer B., Cabrele C. (2018). Reduction of Cancer Cell Viability by Synergistic Combination of Photodynamic Treatment with the Inhibition of the Id Protein Family. J. Photochem. Photobiol. B.

[23] Chang M.H., Pai C.L., Chen Y.C., Yu H.P., Hsu C.Y., Lai P.S. (2018). Enhanced Antitumor Effects of Epidermal Growth Factor Receptor Targetable Cetuximab-Conjugated Polymeric Micelles for Photodynamic Therapy. Nanomaterials.

[24] Tachikawa S., Sato S., Hazama H., Kaneda Y., Awazu K., Nakamura H. (2015). Localization-Dependent Cell-Killing Effects of Protoporphyrin (PPIX)-Lipid Micelles and Liposomes in Photodynamic Therapy. Bioorg. Med. Chem..

[25] Ma Z., Hu P., Guo C., Wang D., Zhang X., Chen M., Wang Q., Sun M., Zeng P., Lu F. (2019). Folate-Mediated and PH-Responsive Chidamide-Bound Micelles Encapsulating Photosensitizers for Tumor-Targeting Photodynamic Therapy. Int. J. Nanomed..

[26] Guirguis M., Bhandari C., Li J., Eroy M., Prajapati S., Margolis R., Shrivastava N., Hoyt K., Hasan T., Obaid G. (2021). Membrane Composition Is a Functional Determinant of NIR-Activable Liposomes in Orthotopic Head and Neck Cancer. Nanophotonics.

